# Supplementary material for: Influence of the Substituent’s Size in the Phosphinate Group on the Conformational Possibilities of Ferrocenylbisphosphinic Acids in the Design of Coordination Polymers and Metal–Organic Frameworks
Source: Int J Mol Sci. 2023 Sep 14;24(18):14087. doi: 10.3390/ijms241814087 (PMC10531850; doi:10.3390/ijms241814087)

checkCIF/PLATON report

You have not supplied any structure factors. As a result the full set of tests cannot be run.

THIS REPORT IS FOR GUIDANCE ONLY. IF USED AS PART OF A REVIEW PROCEDURE FOR PUBLICATION, IT SHOULD NOT REPLACE THE EXPERTISE OF AN EXPERIENCED CRYSTALLOGRAPHIC REFEREE.

No syntax errors found. CIF dictionary Interpreting this report

Datablock: pc

Bond precision: C-C = 0.0030 A Wavelength=0.71073

Cell:	a=8.8000(18)	b=11.320(2)	c=14.480(3)
	alpha=90	beta=106.20(3)	gamma=90
Temperature:	150 K		

	Calculated	Reported
Volume	1385.2(5)	1385.2(5)
Space group	P 2/c	P 2/c
Hall group	-P 2yc	-P 2yc
Moiety formula	C10 H16 N2 Ni O4, C12 H14 Fe O4 P2, 2(H2 O)	C10 H16 N2 Ni O4, C12 H14 Fe O4 P2, 2(H2 O)
Sum formula	C22 H34 Fe N2 Ni O10 P2	C22 H34 Fe N2 Ni O10 P2
Mr	662.99	663.01
Dx,g cm-3	1.590	1.590
Z	2	2
Mu (mm-1)	1.374	1.374
F000	688.0	688.0
F000’	690.06	
h,k,lmax	11,14,18	11,14,15
Nref	3060	2418
Tmin,Tmax	0.885,0.907	0.523,0.746
Tmin’	0.869	

Correction method= # Reported T Limits: Tmin=0.523 Tmax=0.746
AbsCorr = MULTI-SCAN

Data completeness= 0.790 Theta(max)= 27.085

R(reflections)= 0.0328(2393) wR2(reflections)= 0.0906(2418)

S = 1.096 Npar= 194

The following ALERTS were generated. Each ALERT has the format
test-name_ALERT_alert-type_alert-level.
Click on the hyperlinks for more details of the test.

Alert level A

```
PLAT029_ALERT_3_A _diffrn_measured_fraction_theta_full value Low .      0.794 Why?
```

Author Response: the crystal was weakly diffracting needle, but despite the low complet

Alert level C

```
PLAT250_ALERT_2_C Large U3/U1 Ratio for Average U(i,j) Tensor ....        2.1 Note
PLAT250_ALERT_2_C Large U3/U1 Ratio for Average U(i,j) Tensor ....        2.1 Note
```

Alert level G

```
PLAT002_ALERT_2_G Number of Distance or Angle Restraints on AtSite          9 Note
PLAT004_ALERT_5_G Polymeric Structure Found with Maximum Dimension          1 Info
PLAT172_ALERT_4_G The CIF-Embedded .res File Contains DFIX Records          1 Report
PLAT180_ALERT_4_G Check Cell Rounding: # of Values Ending with 0 =          4 Note
PLAT794_ALERT_5_G Tentative Bond Valency for Ni1       (II)      .       1.98 Info
PLAT794_ALERT_5_G Tentative Bond Valency for Fe1       (II)      .       2.14 Info
PLAT802_ALERT_4_G CIF Input Record(s) with more than 80 Characters          1 Info
PLAT860_ALERT_3_G Number of Least-Squares Restraints ............           6 Note
PLAT883_ALERT_1_G No Info/Value for _atom_sites_solution_primary .     Please Do !
PLAT952_ALERT_5_G Calculated (ThMax) and CIF-Reported Lmax Differ.          3 Units
PLAT982_ALERT_1_G The  C-f'=    0.0060 Deviates from  IT-value =       0.0033 Check
PLAT982_ALERT_1_G The Fe-f'=    0.3410 Deviates from  IT-value =       0.3463 Check
PLAT982_ALERT_1_G The  N-f'=    0.0100 Deviates from  IT-value =       0.0061 Check
PLAT982_ALERT_1_G The Ni-f'=    0.2490 Deviates from  IT-value =       0.3393 Check
PLAT982_ALERT_1_G The  O-f'=    0.0180 Deviates from  IT-value =       0.0106 Check
PLAT982_ALERT_1_G The  P-f'=    0.1460 Deviates from  IT-value =       0.1023 Check
PLAT983_ALERT_1_G The  C-f"=    0.0030 Deviates from  IT-Value =       0.0016 Check
PLAT983_ALERT_1_G The Fe-f"=    1.2590 Deviates from  IT-Value =       0.8444 Check
PLAT983_ALERT_1_G The  N-f"=    0.0050 Deviates from  IT-Value =       0.0033 Check
PLAT983_ALERT_1_G The Ni-f"=    1.6440 Deviates from  IT-Value =       1.1124 Check
PLAT983_ALERT_1_G The  O-f"=    0.0100 Deviates from  IT-Value =       0.0060 Check
PLAT983_ALERT_1_G The  P-f"=    0.1480 Deviates from  IT-Value =       0.0942 Check
```

1 **ALERT level A** = Most likely a serious problem - resolve or explain
0 **ALERT level B** = A potentially serious problem, consider carefully
2 **ALERT level C** = Check. Ensure it is not caused by an omission or oversight
22 **ALERT level G** = General information/check it is not something unexpected

13 ALERT type 1 CIF construction/syntax error, inconsistent or missing data
3 ALERT type 2 Indicator that the structure model may be wrong or deficient
2 ALERT type 3 Indicator that the structure quality may be low
3 ALERT type 4 Improvement, methodology, query or suggestion
4 ALERT type 5 Informative message, check

It is advisable to attempt to resolve as many as possible of the alerts in all categories. Often the minor alerts point to easily fixed oversights, errors and omissions in your CIF or refinement strategy, so attention to these fine details can be worthwhile. In order to resolve some of the more serious problems it may be necessary to carry out additional measurements or structure refinements. However, the purpose of your study may justify the reported deviations and the more serious of these should normally be commented upon in the discussion or experimental section of a paper or in the "special_details" fields of the CIF. checkCIF was carefully designed to identify outliers and unusual parameters, but every test has its limitations and alerts that are not important in a particular case may appear. Conversely, the absence of alerts does not guarantee there are no aspects of the results needing attention. It is up to the individual to critically assess their own results and, if necessary, seek expert advice.

Publication of your CIF in IUCr journals

A basic structural check has been run on your CIF. These basic checks will be run on all CIFs submitted for publication in IUCr journals (*Acta Crystallographica*, *Journal of Applied Crystallography*, *Journal of Synchrotron Radiation*); however, if you intend to submit to *Acta Crystallographica Section C* or *E* or *IUCrData*, you should make sure that full publication checks are run on the final version of your CIF prior to submission.

Publication of your CIF in other journals

Please refer to the *Notes for Authors* of the relevant journal for any special instructions relating to CIF submission.

PLATON version of 28/11/2022; check.def file version of 28/11/2022

Datablock pc - ellipsoid plot

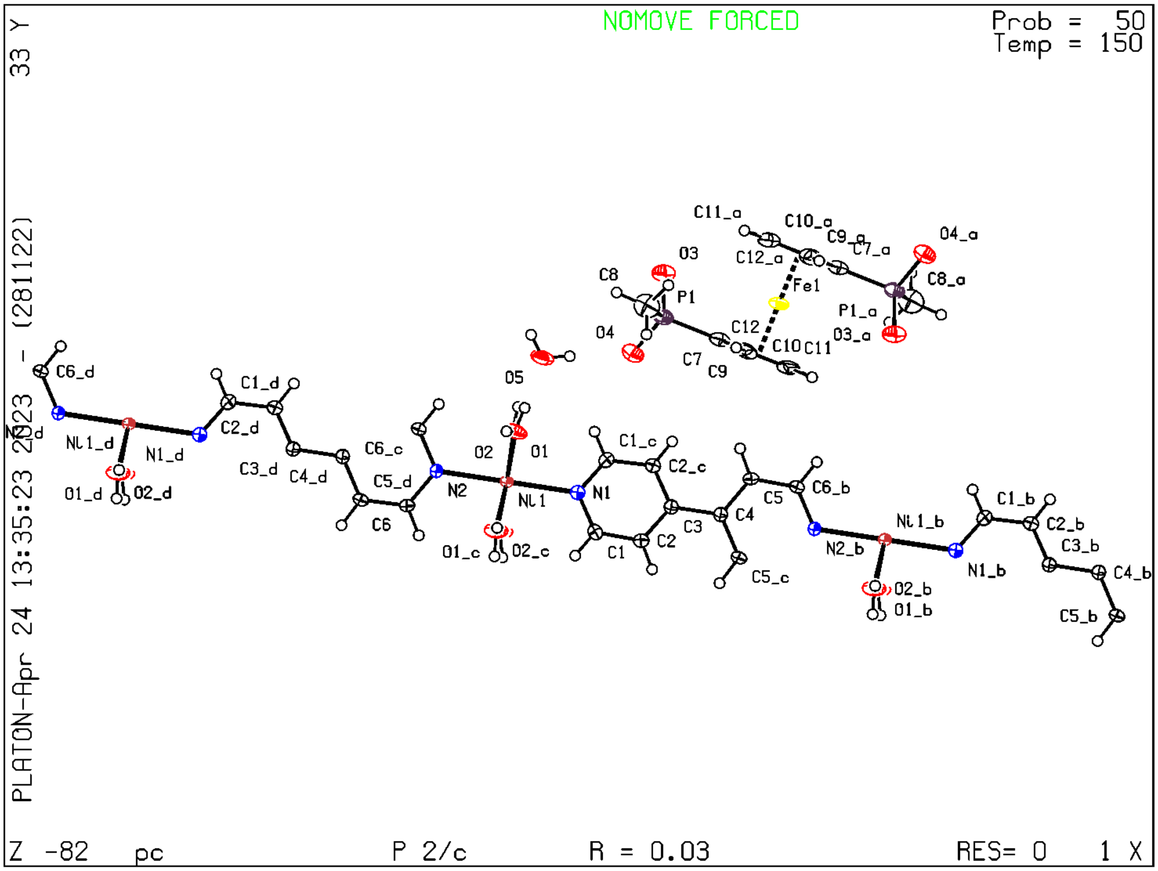

Supplement: Supplementary file 1 [file ijms-24-14087-s001.zip › checkcif_pc.pdf]
