# Supplementary material for: Influence of the Substituent’s Size in the Phosphinate Group on the Conformational Possibilities of Ferrocenylbisphosphinic Acids in the Design of Coordination Polymers and Metal–Organic Frameworks
Source: Int J Mol Sci. 2023 Sep 14;24(18):14087. doi: 10.3390/ijms241814087 (PMC10531850; doi:10.3390/ijms241814087)
Supplement: Supplementary file 1 [file ijms-24-14087-s001.zip › ijms-2579350-supplementary.pdf]

Supporting Information

for

Influence of the Substituent's Size in the Phosphinate Group on the Conformational Possibilities of Ferrocenylbisphosphinic Acids in the Design of Coordination Polymers and Metal–Organic Frameworks

Ruslan P. Shekurov,[1] Mikhail N. Khrizanforov,[1,2*] Ilya A. Bezkishko,[1] Kamil A. Ivshin,[1] Almaz A. Zagidullin,[1,3] Anna A. Lazareva,[1,2] Olga N. Kataeva,[1,2] Vasili A. Miluykov[1]

[1] Arbuzov Institute of Organic and Physical Chemistry, FRC Kazan Scientific Center, Russian Academy of Sciences, 8 Arbuzov Street, 420088 Kazan, Russian Federation,

[2] A.M. Butlerov Chemistry Institute of the Kazan Federal University, 420008 Kazan, Russia

[3] Institute of Fundamental Medicine and Biology, Kazan Federal University, 420008 Kazan, Russia

Table of contents

Table S1. Hydrogen bonds parameters in **10.** 3

Figure S1. Comparison powder TG of coordination polymers **6a-c.** 4

Figure S2. Powder diffraction patterns of coordination polymers **4** based on M(II) cations. 5

Figure S3. Powder diffraction patterns of coordination polymers **5** based on M(II) cations. 6

Figure S4. Powder diffraction patterns of coordination polymers **6** based on M(II) cations. 6

Figure S5. Powder diffraction patterns of coordination polymers **8** based on Ln(III) cations. 7

Figure S6. Powder diffraction patterns of porous coordination polymers **9** based on M(II) cations. 8

Figure S7. Adsorption and desorption isotherms of N_2 (77 K) for **9a**. 9

Figure S8. Adsorption and desorption isotherms of N_2 (77 K) for **5c**. 10

Figure S9. Adsorption and desorption isotherms of H_2O vapor (298 K) and MeOH (298 K) for **5c**. 10

Figure S10. Powder diffraction patterns for **5c** forms. 11

Figure S11. Adsorption and desorption isotherms of N_2 (77 K) for aerogel based on Al(III) ferrocenyl diphosphinate metal–organic framework. 11

Figure S12. Adsorption and desorption isotherms of N_2 (77 K) for aerogel based on nanoporous Fe(III) ferrocenyl diphosphinate metal–organic framework. 12

Scheme S1. Synthesis of coordination polymers with lanthanides (III). 13

Scheme S2. Synthesis of 3D coordination polymer **9**. 13

Figure S13. Thermogravimetric analysis of 3D coordination polymers **9a** and **9b**. 14

References 15

Table S1. Hydrogen bonds parameters in **10**

1	1 O(1)	--H(1A)	..O(5)	[1555.03]	0.79(3)	1.99(3)	2.748(3)	160(3)
2	1 O(1)	--H(1B)	..O(3)	[4565.02]	0.82(3)	1.80(3)	2.618(3)	176(4)
3	1 O(2)	--H(2A)	..O(4)	[3565.02]	0.82(3)	1.82(3)	2.641(3)	176(2)
4	1 O(2)	--H(2B)	..O(5)	[1555.03]	0.80(3)	2.00(3)	2.757(3)	158(4)
5	3 O(5)	--H(5A)	..O(3)	[3665.02]	0.80(3)	1.91(3)	2.701(3)	169(4)
6	3 O(5)	--H(5B)	..O(4)	[1555.02]	0.82(2)	1.93(2)	2.742(3)	171(5)

The thermogravimetric analysis (TGA) of 6a-c

The thermogravimetric analysis (TGA) was carried out on air using a Netzsch STA 409 PC Luxx thermal analyzer, with a heating rate of 5°/min in the temperature region from 20 up to 1200 °C.

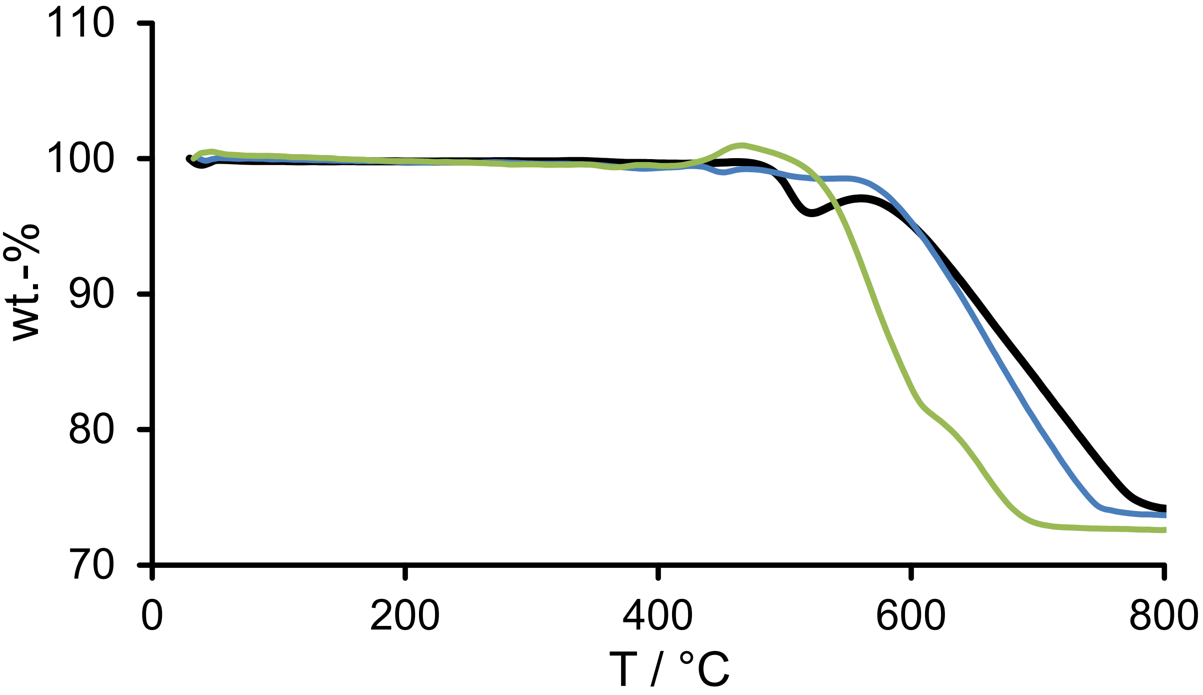

Figure S1. Comparison powder TG of coordination polymers **6a** (Zn- H_2fcd*Me*p, black), **6b** (Co-H_2fcd*Me*p, blue) and **6c** (Mn-H_2fcd*Me*p, green).

Powder X-ray diffraction of CPs.

The X-ray diffraction analysis was performed using an equipment of the Spectral-Analytical Center of FRC Kazan Scientific Center of RAS. Powder X-ray diffraction (PXRD) measurements were performed on a Bruker D8 Advance diffractometer equipped with Vario attachment and Vantec linear PSD, using Cu radiation (40 kV, 40 mA) monochromated with the curved Johansson monochromator (λ Cu $K_{\alpha 1}$ 1.5406 Å) and on a STOE STADI P diffractometer with Cu−$K\alpha_1$ radiation (λ = 1.5405 Å). Room-temperature data were collected in the reflection mode. The samples were loaded on a standard zero diffraction silicon plate, which was spinning (15 rpm) throughout the data collection. Patterns were recorded in the 2Θ range between 3° and 90° , in 0.008° steps, with a step time of 0.1–1.0s. For each sample, several experiments were performed, allowing to control stability of the samples and quality of the experiments. Processing of the obtained data was performed using EVA software packages.

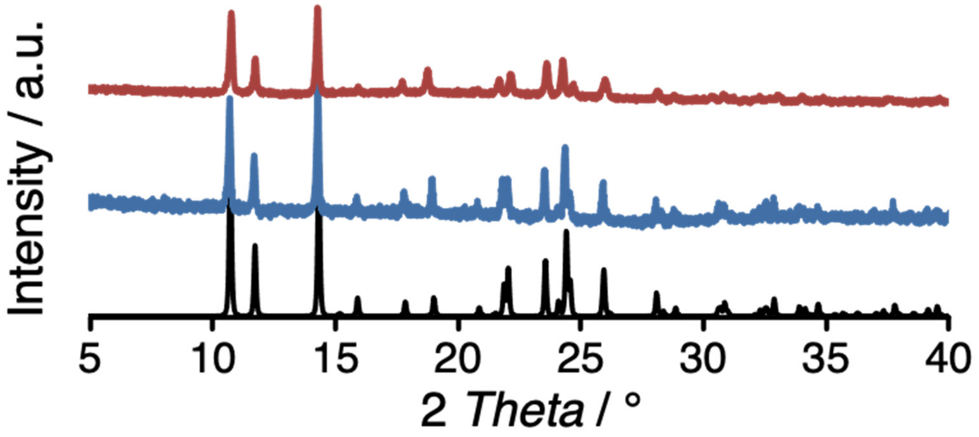

Figure S2. Powder diffraction patterns of coordination polymers **4** based on M(II) cations. From top to bottom: colored curves—the results of measurements of coordination compounds Co and Zn, respectively. Black—simulated spectrum of a phase diffraction pattern from a single crystal X-ray structural analysis of a Zn(II)-based polymer.

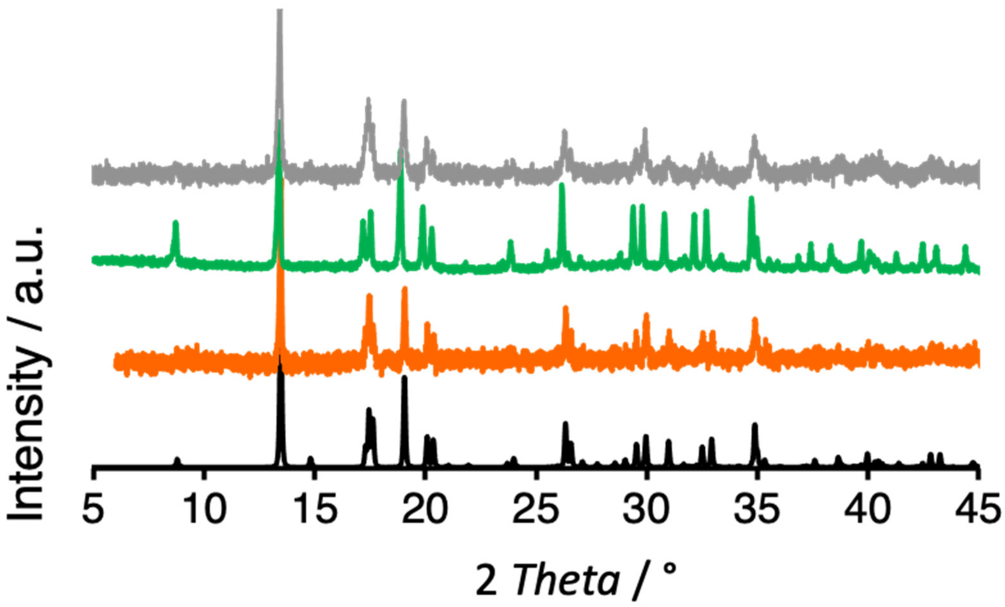

Figure S3. Powder diffraction patterns of coordination polymers **5** based on M(II) cations. From top to bottom: colored curves—the results of measurements of coordination compounds Co, Cd, and Mn, respectively. Black—simulated spectrum of a phase diffraction pattern from a single crystal X-ray structural analysis of a Mn(II)-based polymer.

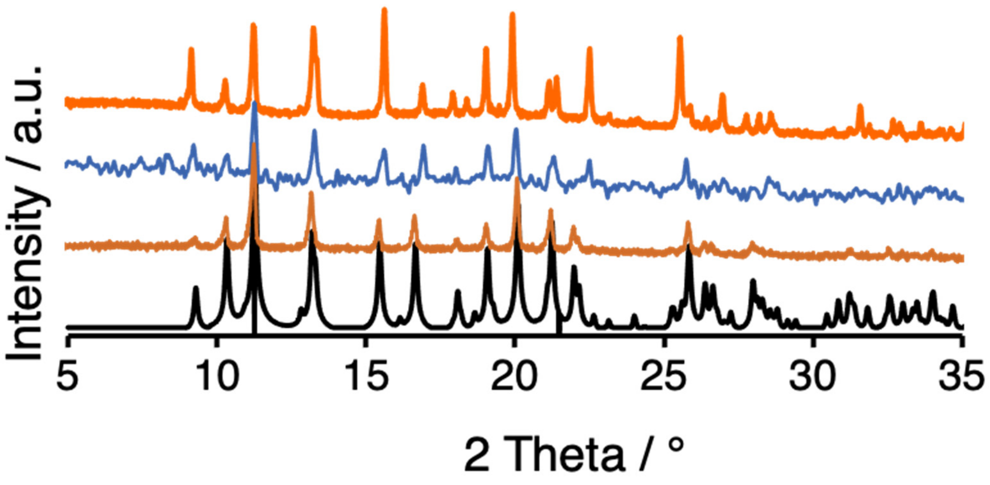

Figure S4. Powder diffraction patterns of coordination polymers **6** based on M(II) cations. From top to bottom: colored curves—the results of measurements of coordination compounds Zn, Co, and Mn, respectively. Black—simulated spectrum of a phase diffraction pattern from a single crystal X-ray structural analysis of a Mn(II)-based polymer.

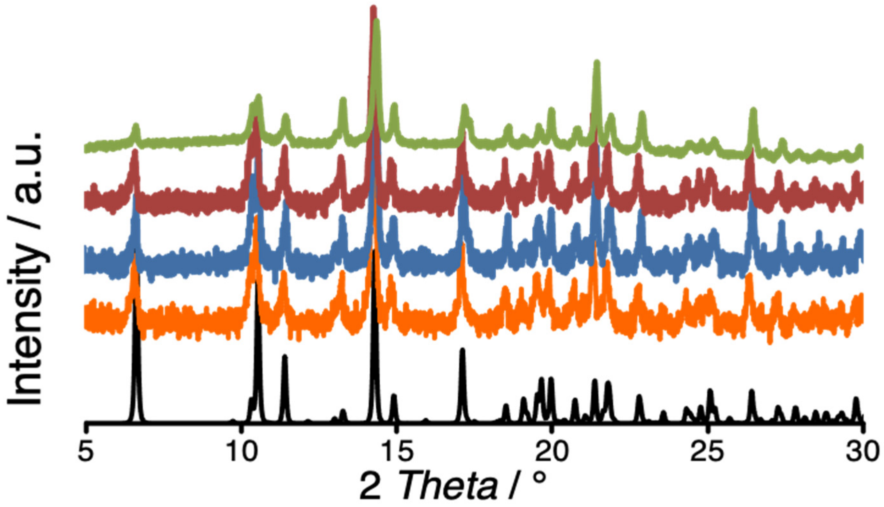

Figure S5. Powder diffraction patterns of coordination polymers **8** based on Ln cations. From top to bottom: colored curves—the results of measurements of coordination compounds Sm, Eu, Dy, and Y, respectively. Black—simulated spectrum of a phase diffraction pattern from a single crystal X-ray structural analysis of a Sm(**III**)-based polymer.

The preliminary drying of polymer **9a** at 150°C under ambient pressure or at 250°C under vacuum does not result in the formation of free pores, as evidenced by the low nitrogen sorption (8 m^2/g) of compound **9a**. Apparently, the activation of the polymer leads to the destruction of its crystalline structure. Therefore, compound 9 can be classified as a first-type porous coordination polymer according to Kitagawa's classification, in which the crystalline structure collapses upon removal of the guest molecule.

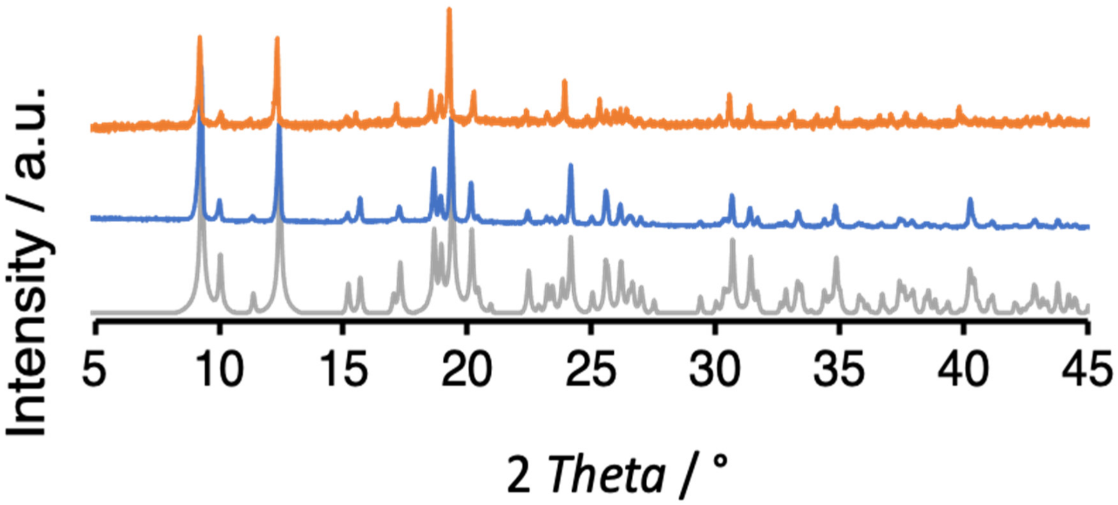

Figure S6. Powder diffraction patterns of porous coordination polymers **9** based on M(II) cations. From top to bottom: colored curves—the results of measurements of coordination compounds Co and Ni, respectively. Grey—simulated spectrum of a phase diffraction pattern from a single crystal X-ray structural analysis of a Ni(II)-based polymer.

Sorption properties of CPs.

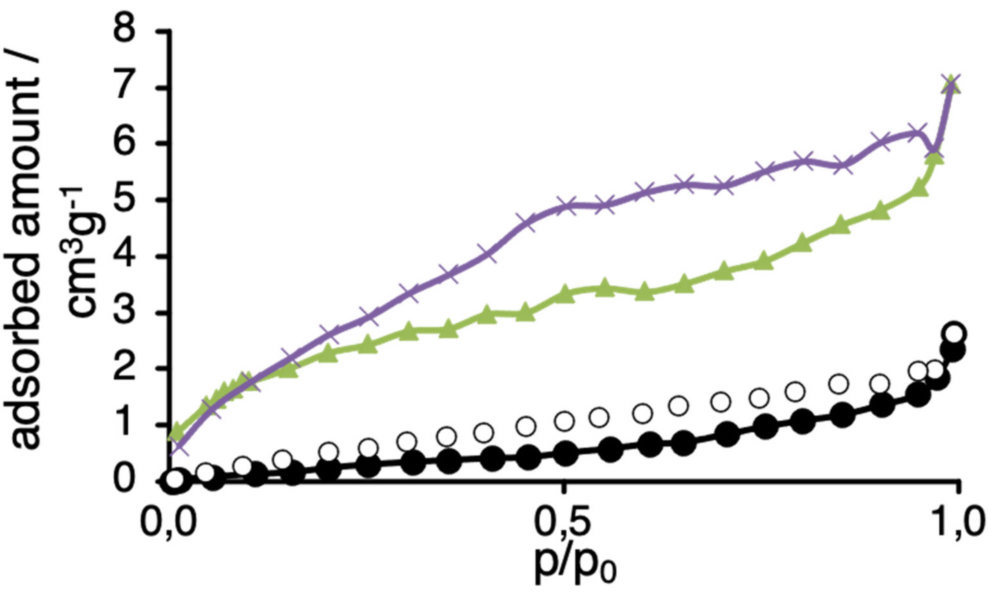

Figure S7. Adsorption and desorption isotherms of N_2 (77 K) for **9a**. From top to bottom: activated polymer after 250C heating and after 150C heating. N_2 cannot enter the cavities of **9a**, and a typical isotherm for non-porous fine powder is obtained. BET surface area 8 m^2/g and pore volume. Open symbols are upon desorption.

Upon heating up to 150°C, the removal of both coordinated and lattice water molecules from **5c** yields a compound that, while impenetrable to nitrogen, exhibits a selective affinity for water over methanol and other solvents at 298 K. This selective water adsorption and desorption process triggers reversible structural changes in the MOF, which also affect its magnetic properties.

Post-dehydration porosity analysis of the compound, conducted via nitrogen physisorption at 77 K, revealed that nitrogen molecules cannot access the dehydrated form of **5c**. Conversely, the compound demonstrates an affinity for water vapor adsorption at 298 K. The uptake trajectory begins with a gradual increase until reaching a p/p0 ratio of 0.8, after which there is a sharp spike in adsorption beyond this pressure. This adsorption trend, transitioning from a Type II to a Type I isotherm, underscores the MOF's flexible response to water adsorption, leading to a structural "gate-opening" modification. The maximal uptake aligns with the presence of four H_2O molecules for each formula unit, which aligns with a 17% weight reduction seen in the TG analysis. Evidently, this activated form of the compound preferentially adsorbs water over other solvents. Notably, the isotherm's desorption pathway deviates from its adsorption trajectory, indicating a wide hysteresis typical of "gate-pressure" MOFs. The entrapped water molecules are not readily desorbed even at diminished relative pressures, necessitating heating in conjunction with evacuation for removal. Additionally, **5c'**s water desorption behavior stands out compared to other flexible porous coordination polymers that house both coordinated and lattice water molecules. While many polymers begin releasing lattice water under standard conditions and others present step-like desorption profiles, **5c** remains notably stable at ambient temperatures, shedding all four water molecules simultaneously when heated.

This water adsorption and subsequent removal mechanism is fully reversible. In a subsequent water vapor adsorption cycle, following an overnight 150°C evacuation, both the volume and pattern of water adsorption remain consistent.

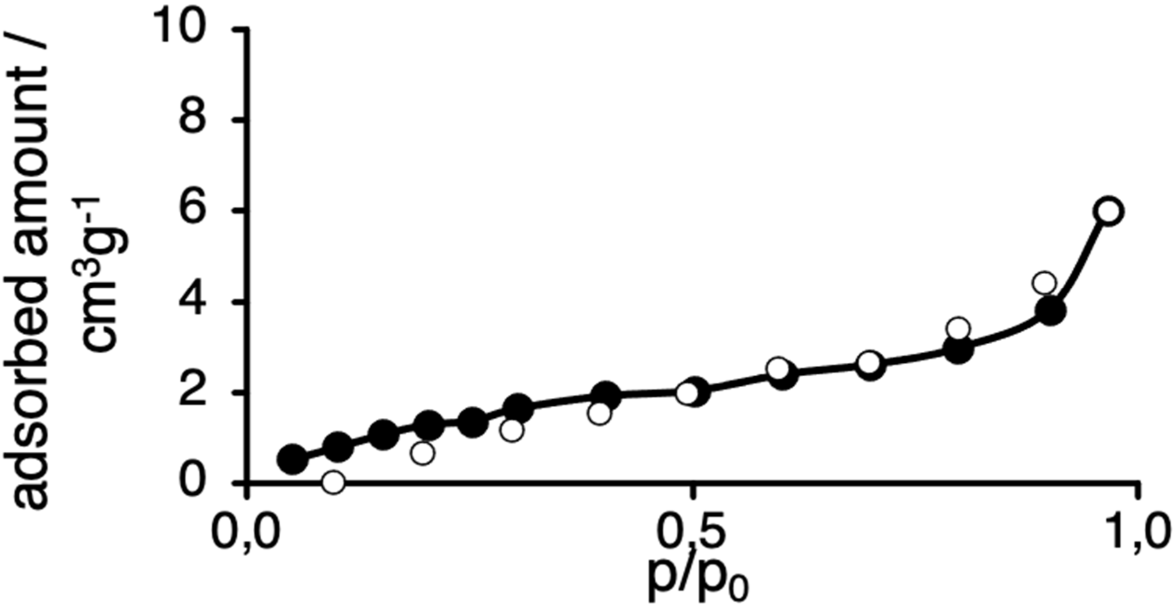

Figure S8. Adsorption and desorption isotherms of N_2 (77 K) for **5c**. N_2 cannot enter the cavities of **5c**, and a typical isotherm for non-porous fine powder is obtained. BET surface area 6 m^2/g and pore volume. Open symbols are upon desorption.

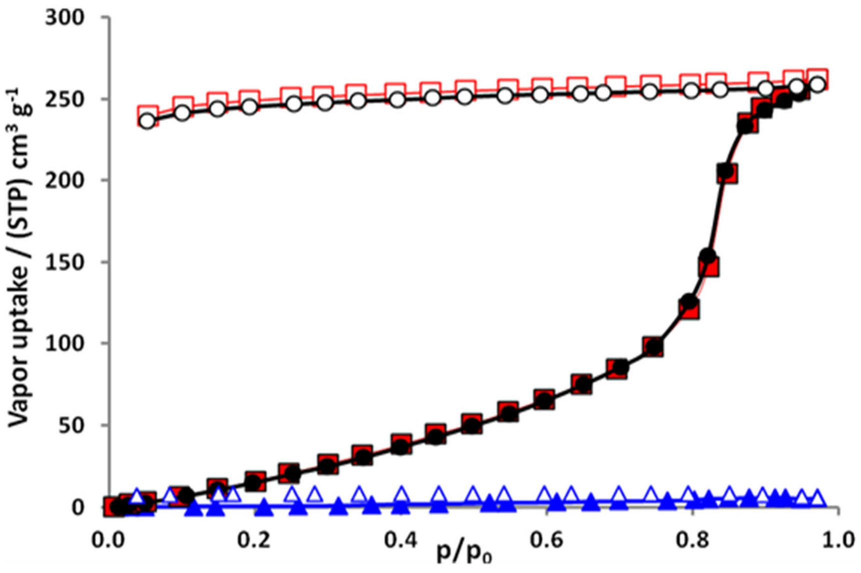

Figure S9. Adsorption (closed symbols) and desorption (open symbols) isotherms of H_2O vapor (298 K) (black circles—cycle 1, and red squares—cycle 2), and MeOH (298 K) (blue triangles) for **5c**. BET surface area 235 m^2/g for H_2O vapor and 8 m^2/g for MeOH.

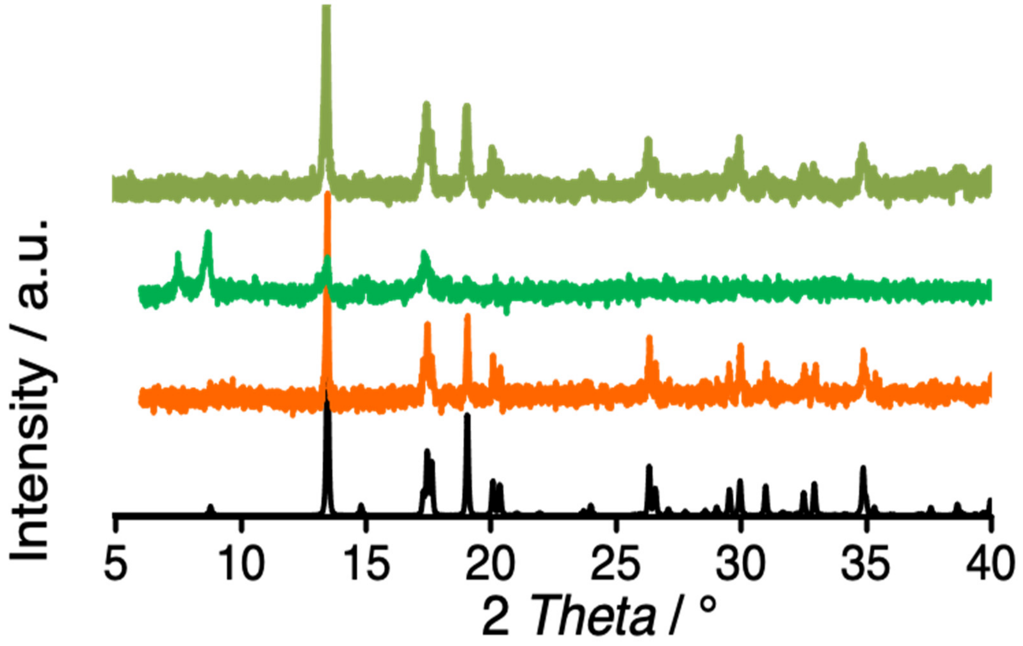

Figure S10. Powder diffraction patterns for **5c** forms. From top to bottom: colored—rehydrated form, dehydrated form, and as-synthesized, respectively. Black—simulated spectrum of a phase diffraction pattern from a single crystal X-ray structural analysis of **5c**.

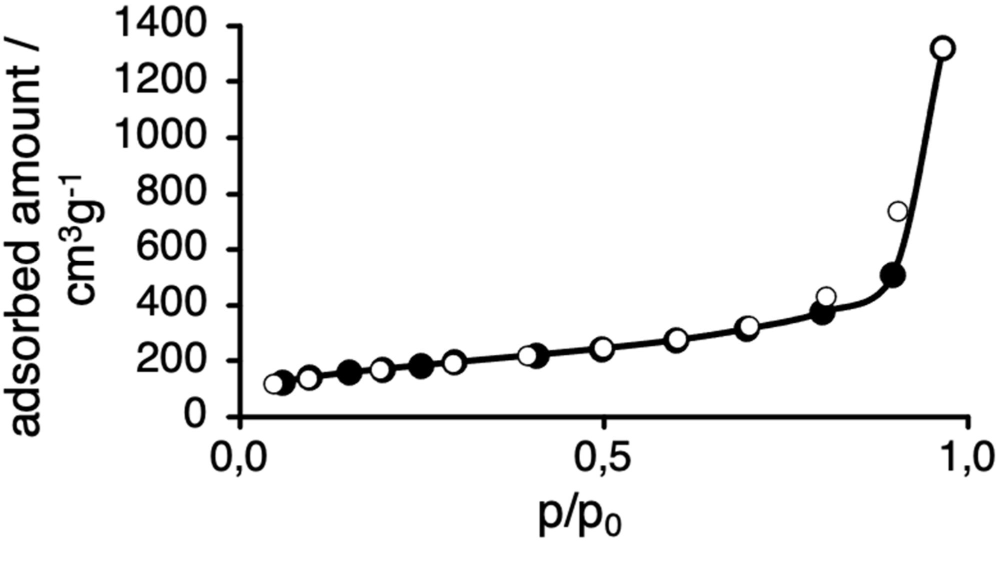

Figure S11. Adsorption and desorption isotherms of N_2 (77 K) for aerogel based on Al(III) ferrocenyl diphosphinate metal–organic framework. N_2 enters the cavities, and a typical isotherm for porous powder is obtained. BET surface area 671 m^2/g and pore volume. Open symbols are upon desorption.

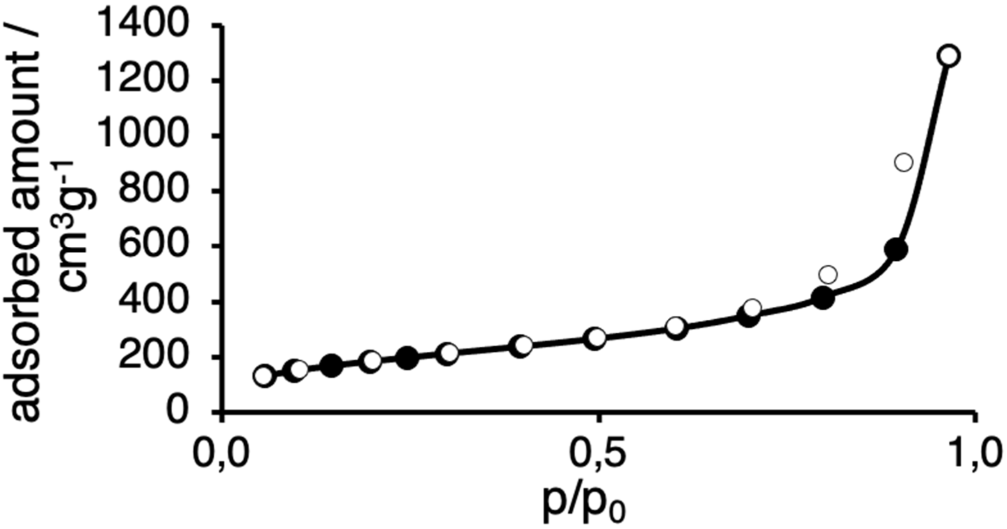

Figure S12. Adsorption and desorption isotherms of N_2 (77 K) for aerogel based on nanoporous Fe(III) ferrocenyl diphosphinate metal–organic framework. N_2 enters the cavities, and a typical isotherm for porous powder is obtained. BET surface area 617 m^2/g and pore volume. Open symbols are upon desorption.

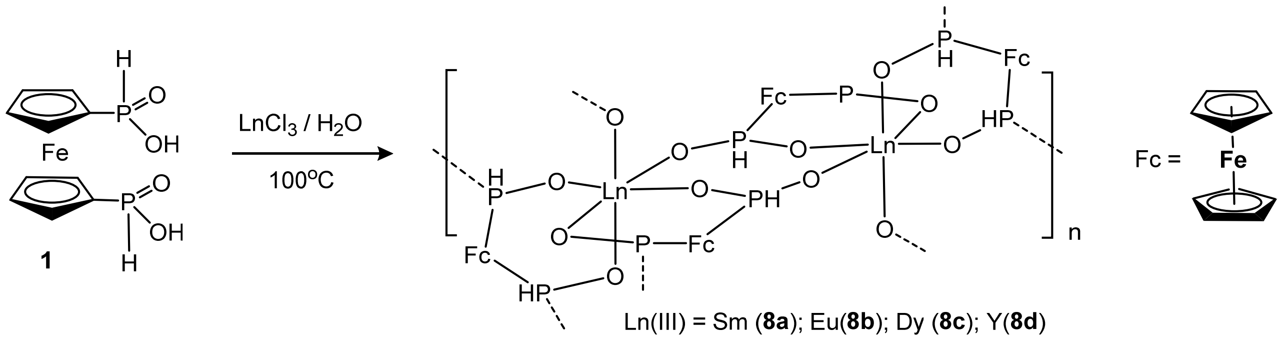

Scheme S1. Synthesis of coordination polymers with lanthanides (**III**).

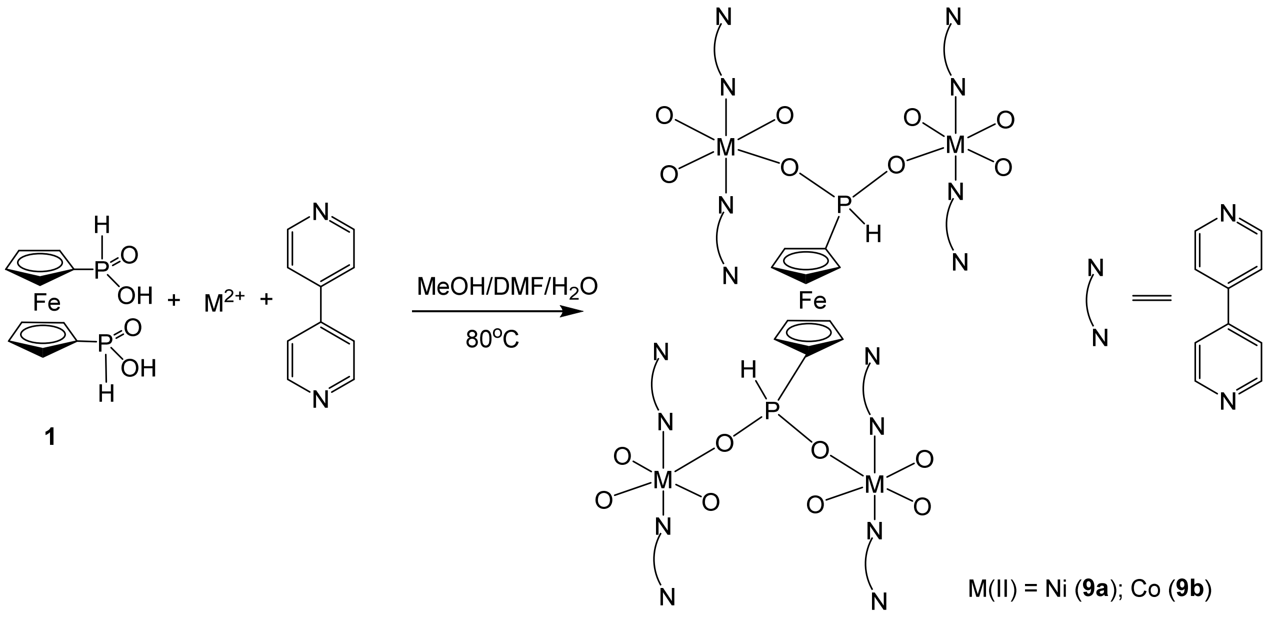

Scheme S2. Synthesis of 3D coordination polymer **9**.

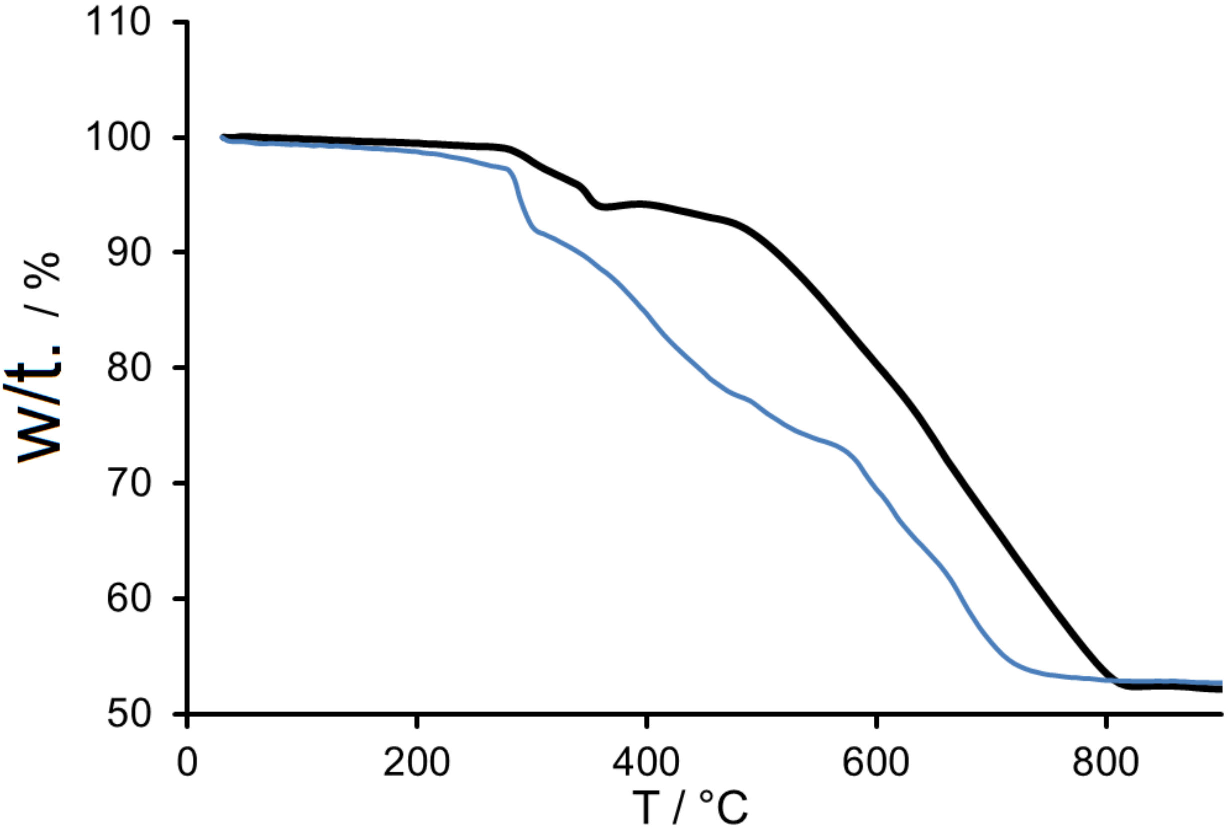

Figure S13. Thermogravimetric analysis of 3D coordination polymers **9a** (black curve) and **9b** (gray curve).

References

[1] Bruker. APEX3 Crystallography Software Suite; Bruker AXS Inc.: Madison, WI, USA, 2016.Bruker. Area detector control and integration software. Version 5.x. In: SMART

[2] Bruker. SAINT Crystallography Software Suite; Bruker AXS Inc.: Madison, WI, USA, 2016.

[3] Krause, L.; Herbst-Irmer, R.; Sheldrick, G.M.; Stalke, D. Comparison of silver and molybdenum microfocus X-ray sources for single-crystal structure determination. J. Appl. Crystallogr., 2015, 48, 3–10.

[4] Sheldrick, G. M. A Short History of SHELX. Acta Crystallogr., Sect. A, 2008, 64, 112–122.

[5] Shekurov, R.; Miluykov, V.; Islamov, D.; Krivolapov, D.; Kataeva, O.; Gerasimova, T.; Katsyuba, S.; Nasybullina, G.; Yanilkin, V.; Sinyashin, O. Synthesis and structure of ferrocenylphosphinic acids. J. Organomet. Chem. 2014, 766, 40–48.

[6] Shekurov, R.P.; Tufatullin, A.I.; Milyukov, V.A.; Kataeva, O.N.; Sinyashin, O.G. Supramolecular architecture of diammonium ferrocene-1,1’-diyldiphosphinates. Russ. Chem. Bull. 2014, 63, 178–181.

[7].Shekurov, R.; Khrizanforov, M.; Ivshin, K.; Miluykov, V.; Budnikova, Y.; Kataeva, O. Supramolecular architecture of diam-monium ferrocene-1,1′-diyldi(methylphosphinate). J. Organomet. Chem. 2019, 904, 121004.

[8].Shekurov, R.; Khrizanforova, V.; Gilmanova, L.; Khrizanforov, M.; Miluykov, V.; Kataeva, O.; Yamaleeva, Z.; Burganov, T.; Gerasimova, T.; Khamatgalimov, A.; Katsyuba, S.; Kovalenko, V.; Krupskaya, Y.; Kataev, V.; Büchner, B.; Bon, V.; Senkovska, I.; Kaskel, S.; Gubaidullin, A.; Sinyashin, O.; Budnikova, Y. Zn and Co Redox Active Coordination Polymers Based on Fer-rocene-containing Diphosphinate Ligand as Efficient Electrocatalysts for Hydrogen Evolution Reaction. Dalton Trans. 2019, 48, 3601–3609.

[9] Shekurov, R.; Miluykov, V.; Kataeva, O.; Krivolapov, D.; Sinyashin, O.; Gerasimova, T.; Katsyuba, S.; Kovalenko, V.; Krupskaya, Y.; Kataev, V.; Büchner, B.; Senkovska, I.; Kaskel, S. Reversible water-induced structural and magnetic transfor-mations and selective water adsorption properties of poly (manganese 1,1′-ferrocenediyl-bis (H-phosphinate)). Cryst. Growth Des. 2016, 16 (9), 5084–5090.

[10] Shekurov, R.P.; Gilmanova, L.H.; Miluykov V.A. New porous Fe(III)-based ferrocene-containing diphosphinate. Phosphorus, Sulfur, Silicon Relat. Elem. 2019, 194 (10), 1007–1009.

[11] Khrizanforova, V.; Shekurov, R.; Miluykov, V.; Khrizanforov, M.; Bon, V.; Kaskel, S.; Gubaidullin, A.; Sinyashin O.; Budnikova, Y. 3D Ni and Co Redox-Active Metal-Organic Frameworks Based on Ferrocenyl Diphosphinate and 4,4’-Bipyridine Ligands as Efficient Electrocatalysts for Hydrogen Evolution Reaction. Dalton Trans. 2020, 49, 2794–2802.

[12] Maji, T.K.; Kitagawa S, Chemistry of porous coordination polymers. Pure Appl. Chem. 2007, 79(12), 2155–2177.
